# Polymorphism of *MTHFR* 1298A>C in relation to adverse pregnancy outcomes in Chinese populations†

**DOI:** 10.1002/mgg3.642

**Published:** 2019-03-22

**Authors:** Hui Mo, Meng Rao, Gang Wang, Yan‐Xi Long, Hua‐Wei Wang, Li Tang

**Affiliations:** ^1^ Department of Reproduction and Genetics Kunming Medical University First Affiliated Hospital Kunming, Yunnan China

**Keywords:** folic acid, *MTHFR*, polymorphism, pregnancy outcome

## Abstract

**Background:**

Adverse pregnancy outcomes (APOs) are involved in a series of abnormal pregnancies like embryo growth arrest, spontaneous abortion, premature birth, stillbirth, fetal malformation, birth defects and other pathological pregnancy, and childbirth complications. Polymorphism of methylenetetrahydrofolate reductase gene (*MTHFR*, 607093) is one of the main genetic causes of APO. However, there is still debate on whether *MTHFR *1298A>C, rs1801131, polymorphism is related to APO. For the lack of extensive research in the Chinese population at present, the study aim to investigate the relationship between *MTHFR* 1298A>C polymorphism with APO through a large number of data.

**Methods:**

A total of 241 samples from patients with APO and 117 healthy controls in Yunnan province were used for *MTHFR *gene polymorphism analysis, with double fluorescence quantitative polymerase chain reaction (PCR). In consideration of the low frequency of *MTHFR *1298C/C genotype, which might affect the statistic results, further datasets of *MTHFR* 1298A>C polymorphism were collected from literature and analyzed.

**Results:**

No statistical difference was detected in the frequency of *MTHFR*1298A>C polymorphism between two groups in Yunnan. Our data showed that the frequency of *MTHFR* 1298A/A genotype had the decreasing tendency among Chinese population from northern to southern, as well as eastern to western of China. The frequency of *MTHFR* 1298A/C and 1298C/C genotypes had the adverse tendency. The frequency of *MTHFR *1298C/C genotype was significantly different between two groups in Chinese populations. The significant difference was also observed in the frequency of *MTHFR* 1298C/C polymorphism between two groups from central China and southern China.

**Conclusion:**

In summary, our data showed that *MTHFR *1298C/C genotype was one of the important genetic factors of APO in China.

## 1 INTRODUCTION

Adverse pregnancy outcomes (APOs) are involved in a series of abnormal pregnancies like embryo growth arrest, spontaneous abortion, premature birth, stillbirth, fetal malformation, birth defects and other pathological pregnancy, and childbirth complications, with a prevalence of 10%–15% in China (Raisanen, Georgiadis, Harju, Keski, & Heinonen, 2012). At present, postpone of childbearing age, biological factors (e.g. viruses, bacteria and parasitic infections, etc.), and immune factors, as well as the increasing life pressure, may cause the occurrence of APO (Hu, 2013). Moreover, genetic factor is also an important aspect related to APO. Polymorphism of methylenetetrahydrofolate reductase (*MTHFR*) gene is deemed as one of the major genetic factors causing APO.

*MTHFR* is a key enzyme in the folic acid metabolic pathway, its encoding gene is located in chromosome 1p36.3, which is the site of multiple single nucleotide polymorphism. The mutation of *MTHFR* 677C>T and 1298A>C genes induce the conversion of valine and glutamic acid from alanine, respectively, resulting in the decrease in 5,10‐methylenetetrahydrofolate reductase activity, subsquently reduce the capacity regarding the conversion of 5‐methylenetetrahydrofolate from 5,10‐methylenetetrahydrofolate, as well as decreasing the folic acid utilization. Moreover, the above mutations also increase the homocysteine (*HCY*) concentration and thereafter induce vascular endothelial damage and dysfunction, destroy the coagulation and fibrinolytic system, subsquently lead to a hypercoagulable status, ultimately result in recurrent abortion, fetal growth restriction, and stillbirth (Nair, Anuradha, Rajender, & Singh, 2013).

*MTHFR* 677C>T and 1298A>C locus have long been investigated (Chedraui et al., 2015; Li, Chen, Guo, & Qiang, 2015; Stangler et al., 2013). A number of studies have shown that polymorphism of *MTHFR* 677C>T is one of the main causes of APO (Liu, Yan, & Yang, 2015; Sun, Wang, & Feng, 2017; Wang et al.., 2010). However, there is debate on whether *MTHFR* 1298A>C polymorphism is related to APO. Recent observations have reported that *MTHFR* 1298A>C polymorphism is one of the factors leading to APO (Chedraui et al., 2014; Li, Wu, He, Lv, & Fu, 2015), with other studies being contrary to this conclusion (Gao et al., 2015; Li, 2014; Xie et.al., 2016). Small sample size and the relatively low frequency distribution of *MTHFR* 1298C/C genotype may contribute to the inconsistent conclusions.

To investigate the relationship between *MTHFR* 1298A>C polymorphism and APO, we collected 241 samples from patients with APO in Yunnan province, southwest of China. Furthermore, the relevant articles on *MTHFR* 1298A>C polymorphism from the China National Knowledge Internet (CNKI) database of Chinese were gathered. The purpose of this study is to estimate the distribution of *MTHFR* 1298A>C gene polymorphism in China, and to affirm the relationship between *MTHFR* 1298A>C polymorphism and APO, and to provide a scientific basis for the diagnosis and treatment of the patients with APO, from the perspective of *MTHFR* polymorphism.

## 2 MATERIAL AND METHODS

### 2.1 Ethical compliance

The study protocol was approved by the Ethics Committee of the First Affiliated Hospital of Kunming Medical University, and informed consents were obtained from all participants who were enrolled in this study. There is no conflict of interest in the study.

### 2.2 Participants

This study was conducted in the First Affiliated Hospital of Kunming Medical University from January to December in 2017 from Yunnan province. A total of 241 samples from patients with APO and 117 healthy controls were used for *MTHFR *gene polymorphism analysis.

### 2.3 Samples collection

The samples were collected by the criteria of the eighth edition for Obstetrics and Gynecology (Xie & Gou, 2014). Inclusion criteria: The patients who have prior history of the spontaneous abortion, missed abortion, fetal malformation, stillbirth, neonatal death, and other adverse pregnancy outcome. Exclusion criteria: Maternal pregnancy patients with viral infection, radiation, smoking, drinking, and drug history, the chromosomal karyotype of the peripheral blood of the couple was abnormal, anatomic malformation was diagnosed by gynecologic, B‐ultrasound, hysterography, abnormal endocrine function including diabetes history, abnormal thyroid function and soon, vein history of embolism, liver and kidney acute and chronic diseases.

A total of 117 healthy persons without family history were matched for the same sex and age from the same hospital in the same period as control group.

### 2.4 DNA extraction and polymerase chain reaction (PCR)

Venous blood specimen was acquired from each participant in the morning. Blood samples were centrifuged at 1,000 *g* for 5 min and the upper serum was collected. Genomic DNA was extracted and purified from blood samples using the genomic DNA Kit (Guangzhou Fei Yang Bioengineering Co. Ltd.), and stored at −20°C.

The dual fluorescence quantitative PCR was used to test the genotype of *MTHFR* 1298A>C, (rs1801131, 607093.0004). The total volume of each reaction system was 6 l, which contains the fluorophore premixed liquid 5 l, purified water 1 l, where reaction condition is 95°C for 10 min to activate fluorescent groups, following by 40 cycles of amplification (95°C for 15 s, 60°C for 40 s, the forward primer sequence was AGAGCAAGTCCCCCAAGGA, the reverse primer sequence was GAGGTAAAGAACGAAGACTTCAAAGAC), cooling at room temperature for 1 min. After the reaction, the ABI step one Plus fluorescent quantitative PCR was used to read the endpoint fluorescence in the sample hole, and the analysis software was served to determine the genotype results of each sample.

### 2.5 Database search

We further searched the CNKI database to collect related articles on *MTHFR* 1298A>C polymorphism. Literatures were eligible if the following criteria were met: the study of *MTHFR* 1298A>C gene polymorphism and APO, patients with APO as case group, healthy persons as control group, the literature is Chinese or English. Exclusion criteria: Non correlation study, incomplete data or unclear description, the results do not conform to the standard literature data, the most complete literature on the selection of duplicated or overlapping documents.

According to the inclusion and exclusion criteria, the retrieved documents were collated and eventually included 25 Chinese and English standard documents. There were 3,722 cases in the cumulative case group and 5,179 cases in the control group.

### 2.6 Data analysis

SPSS 17.0 software was used for data statistical analysis. The genotype frequency was estimated using simple proportions, and the chi‐square test was applied to compare the genotype frequencies between groups. The geographic origin of samples were shown by the black bold circles corresponding to their respective different locations in the map. The spatial‐frequency distributions were created by using the Kriging algorithm of the Surfer 8.0 package. The difference was considered statistically significant when a *p* value was <0.05.

## 3 RESULT

### 3.1 Frequencies of MTHFR 1298A>C polymorphism in Yunnan

The result of the frequency distribution of *MTHFR* 1298A>C polymorphism in Yunnan was presented in Table 1. We found that the genotype frequency of *MTHFR*1298A/A, 1298A/C, and 1298C/C were 157 (65.1%), 73 (30.3%), and 11 (4.6%), respectively in patients with APO. Meanwhile the genotype frequency of *MTHFR* 1298A/A, 1298A/C, and 1298C/C were 82 (70.1%), 29 (24.8%), and 6 (5.1%), respectively in the healthy controls. No significant difference in the frequency of *MTHFR* 1298A>C polymorphism between two groups was found (all *p* > 0.05).

**Table 1:** Frequency comparison of *MTHFR* 1298A>C polymorphism between two groups in Yunnan

	*n*	A/A	A/C	C/C
Experimental group	241	157 (65.1%)	73 (30.3%)	11 (4.6%)
Control group	117	82 (70.1%)	29 (24.8%)	6 (5.1%)
χ^2^		0.005	0.003	0.215
*p*		0.943	0.957	0.643

MTHFR: methylenetetrahydrofolate reductase

### 3.2 Specimen source of MTHFR 1298A>C polymorphism in China

**Figure 1:**
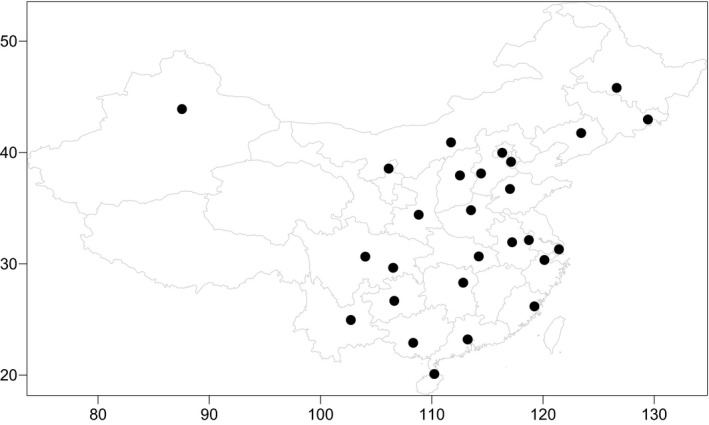
The sample source of MTHFR 1298A>C polymorphism were represented by black bold circles. Specimens were mainly derived from the east China, central China, and southern China, a small number of specimens were from the north, northwest, and southwest of China. MTHFR: methylenetetrahydrofolate reductase

Due to the small sample size and the low frequency of *MTHFR* 1298C/C genotype between two groups from Yunnan province, we collected the related articles on the *MTHFR* 1298A>C polymorphism from the CNKI database. Figure 1 showed the sample source of *MTHFR* 1298A>C polymorphism according to published literatures, where was represented by black bold circles in this study, specimens were mainly derived from the east China, central China, and southern China in this study. However a small number of specimens were from the north, northwest, and southwest of China (Figure 1).

### 3.3 Frequency distribution of MTHFR 1298A>C polymorphism in China

**Figure 2:**
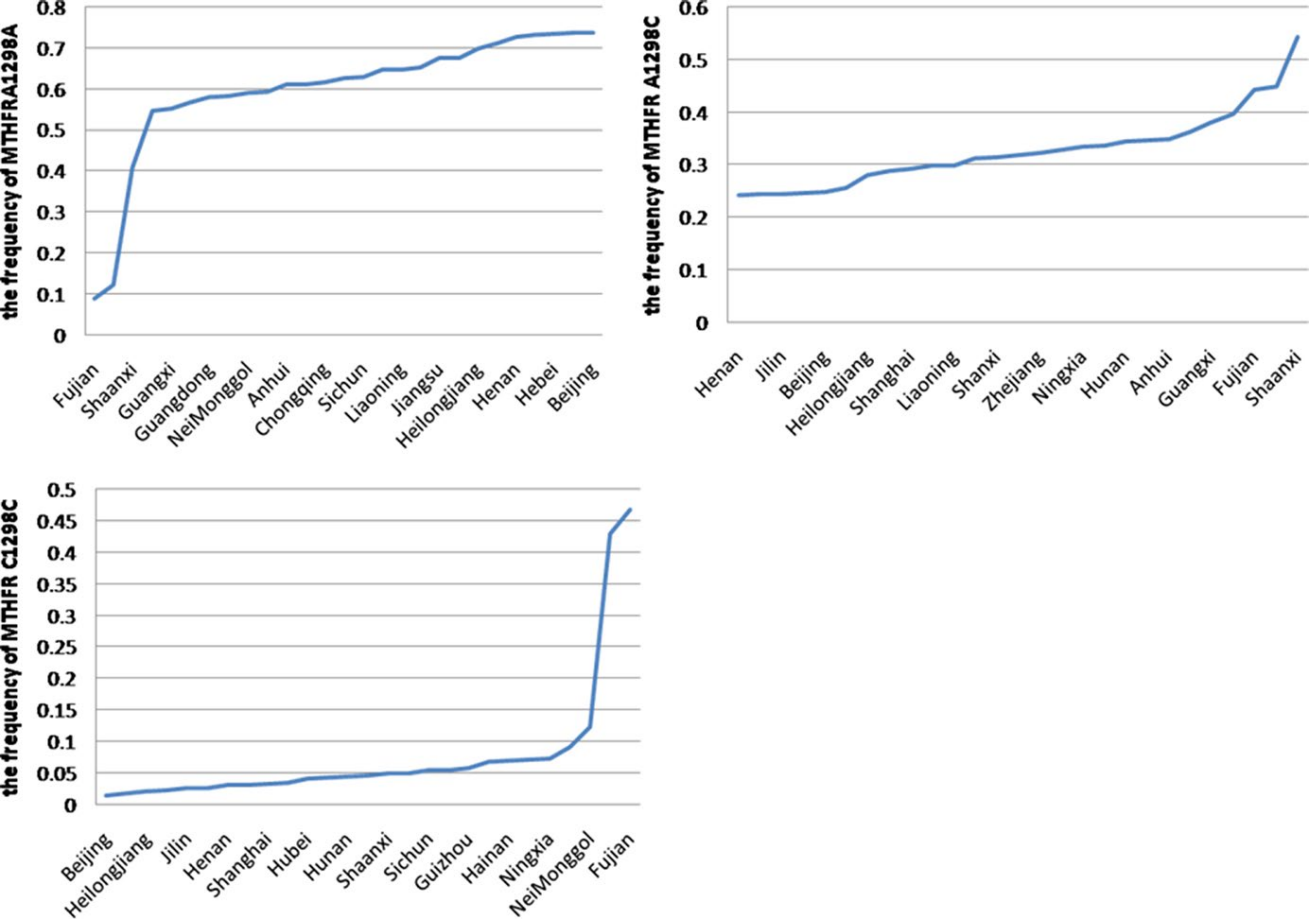
The frequency distribution of MTHFR 1298A>C polymorphism in China. The frequency of *MTHFR* 1298A/A genotype was the lowest in Fujian (9%), whereas the frequency was the highest in Beijing (73.6%). As for *MTHFR*1298A/C, we found that the lowest (24.1%) and the highest (54.3%) frequencies were in Henan and Shanxi respectively. In contrast to MTHFR 1298A/A, the frequency of MTHFR 1298C/C genotype was found the lowest (1.55%) in Beijing and the highest (46.7%) in Fujian. MTHFR: methylenetetrahydrofolate reductase

We furthermore analyzed the frequency distribution of *MTHFR* 1298A>C polymorphism in China, as shown in Figure 2. The frequency of *MTHFR* 1298A/A genotype was 9% in Fujian, as the lowest frequency, whereas the frequency was 73.6% in Beijing, as the highest frequency. As for *MTHFR*1298A/C, we found the lowest (24.1%) and the highest (54.3%) frequencies were in Henan and Shanxi, respectively. In contrast to *MTHFR* 1298A/A, the frequency of *MTHFR* 1298C/C genotype was the lowest (1.55%) in Beijing, yet the frequency of *MTHFR* 1298C/C genotype was the highest (46.7%) in Fujian.

### 3.4 Distribution characteristics of MTHFR 1298A>C polymorphism in China

**Figure 3:**
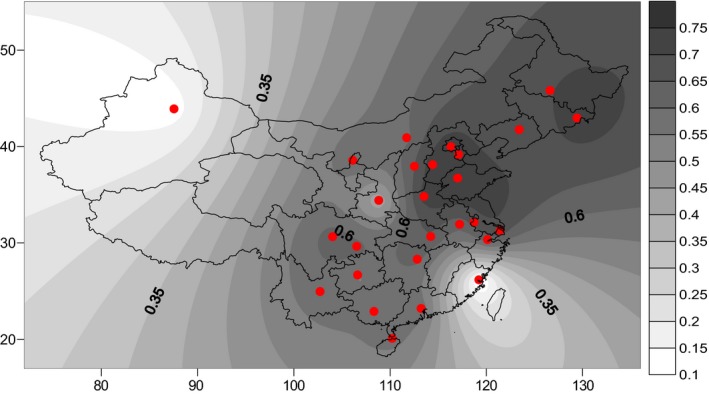
The frequency of the MTHFR 1298A/A genotype was obviously reduced from the north to south and from east to west of China. MTHFR: methylenetetrahydrofolate reductase

**Figure 4:**
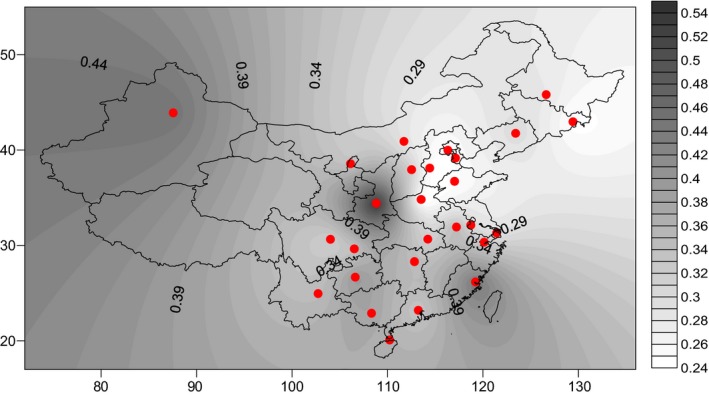
The frequency of MTHFR 1298A/C genotypes had the increasing tendency from north to south and from east to west of China. MTHFR: methylenetetrahydrofolate reductase

**Figure 5:**
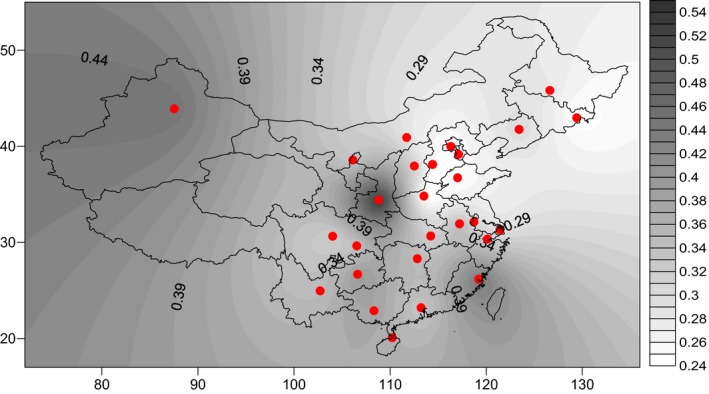
The frequency of MTHFR 1298C/C genotypes had the increasing tendency from north to south and from east to west of China. MTHFR: methylenetetrahydrofolate reductase

An obvious geographical distribution pattern of *MTHFR* 1298A>C polymorphism in China was observed. The result indicated that the frequency of *MTHFR*A 1298A/A genotype was obviously reduced from the north to south and from east to west of China (Figure 3). By contrast, the frequency of *MTHFR* 1298A/C and 1298C/C genotypes has the increasing tendency from north to south and from east to west (Figures 4 and 5).

### 3.5 Frequencies of MTHFR 1298A>C polymorphism in different regions of China

In order to remove the regional difference of *MTHFR* 1298A>C polymorphism, the study divided China into six regions by the geographical region. Table 2 showed that no significant difference was observed in the frequency of *MTHFR* 1298A/C genotype between two groups in northern China, eastern China, northwestern China, and southwestern China (all *p* > 0.05). In central China and southern China, no significant difference was found in the frequency of both *MTHFR* 1298A/A and 1298A/C genotypes (both *p* > 0.05), yet there was a significant difference in 1298C/C genotype between two groups (*p* < 0.05).

**Table 2:** Frequency comparison of *MTHFR* 1298A>C polymorphism between two groups in different regions of China

Zone	*n*	A/A	A/C	C/C
North China				
Experimental group	759	488 (64.3%)	240 (31.6%)	31 (4.1%)
Control group	659	472 (71.6%)	163 (24.7%)	24 (3.7%)
*p*		0.197	0.032	0.679
East China				
Experimental group	1,108	691 (62.4%)	369 (33.3%)	48 (4.3%)
Control group	963	606 (62.9%)	330 (34.3%)	27 (2.8%)
*p*		0.804	0.328	0.073
Central China				
Experimental group	737	398 (54.0%)	233 (31.6%)	106 (14.4%)
Control group	1,455	898 (61.7%)	476 (32.7%)	81 (5.6%)
*p*		0.076	0.710	0.000
South China				
Experimental group	705	404 (57.3%)	252 (35.7%)	49 (7.0%)
Control group	994	594 (59.8%)	370 (37.2%)	30 (3.0%)
*p*		0.605	0.671	0.000
Northwest region				
Experimental group	33	16 (48.5%)	15 (45.5%)	2 (6.0%)
Control group	582	285 (49.0%)	245 (42.1%)	52 (8.9%)
*p*		0.975	0.811	0.599
Southwest region				
Experimental group	380	236 (62.1%)	123 (32.4%)	21 (5.5%)
Control group	273	167 (61.2%)	93 (34.0%)	13 (4.8%)
*p*		0.906	0.747	0.680

MTHFR: methylenetetrahydrofolate reductase

### 3.6 Frequencies of MTHFR 1298A>C polymorphism in China

The study showed that the genotype frequency of *MTHFR* 1298A/A, 1298A/C, and 1298C/C with APO were 2,233 (60.0%), 1,232 (33.1%), and 257 (6.9%), respectively. The genotype frequency of *MTHFR *1298A/A, 1298A/C, and 1298C/C with healthy controls were 3,172 (61.2%), 1758 (34.0%), and 249 (4.8%), respectively (Table 3). The frequency distribution of *MTHFR* 1298A>C polymorphism with APO was similar with that of the healthy controls. There was no statistical difference in the frequency distribution of 1298A/A and 1298A/C genotypes between two groups in China (*p* > 0.05). However significant difference were observed in the frequency distribution of *MTHFR* 1298C/C genotype between two groups (*p* = 0.000).

**Table 3:** Frequency comparison of *MTHFR* 1298A>C polymorphism between two groups in China

	*n*	A/A	A/C	C/C
Experimental group	3,722	2,233 (60.0%)	1,232 (33.1%)	257 (6.9%)
Control group	5,179	3,172 (61.2%)	1,758 (34.0%)	249 (4.8%)
χ^2^		0.349	0.344	15.801
*p*		0.555	0.557	0.000

MTHFR: methylenetetrahydrofolate reductase

## 4 DISCUSSION

Previous studies have demonstrated that the enzyme activity of *MTHFR* 1298A/A and 1298A/C gene was normal and the enzyme activity of *MTHFR* 1298C/C gene was reduced, further decline of enzyme activity influenced the level of folic acid and *HCY* concentration, ultimately increase the risk of APO (Chedraui et al., 2015). We found no significant difference in the frequency of *MTHFR* 1298C/C genotype between patients with APO and healthy controls in Yunnan. However the significant difference was observed with the frequency of *MTHFR* 1298C/C genotypes between two groups in China (*p* < 0.05). The results suggested that the polymorphism of the *MTHFR* 1298A>C was closely related with APO. The small sample size and the relatively low frequency of *MTHFR* 1298C/C genotype might lead to quite different conclusions. Furthermore, we finally draw accurate conclusion by the extensive data statistics in Chinese population.

As a member of vitamin B family, folic acid needs to be activated by folic acid reductase so as to play its physiological activity. As a donor of a single carbon unit in the body, folic acid participates in the methylation of genes and the synthesis of deoxyribonucleic acid. Furthermore it can reduce the concentration of *HCY*, improve the blood supply of placenta, and decrease the risk of thrombus formation. Therefore, effective supplementation of the folic acid decrease the risk of abortion, cardiovascular disease and cancer, etc (Tamura & Picciano, 2006). Folic acid could not be synthesized in human body and has to be supplied from daily food. Consequently, people carrying C1298C gene should be properly supplemented with folic acid to prevent APO.

As showed in the Figures 1, 2, 3, 4, 5, the frequency distribution of the *MTHFR* 1298A>C polymorphism has regional differences. We observed a significant reduction in the frequency distribution of *MTHFR*1298A/A gene from north to south and from east to west of China (Figure 3). The frequency distribution of *MTHFR *1298A/C and 1298C/C genes was opposite to that of *MTHFR* 1298A/A gene, which was higher and higher from north to south and from east to west of China (Figures 4 and 5). The results indicated the frequency distribution of *MTHFR*1298C/C genotype was concentrated in western and southern China (Figure 2). Obviously, the unreasonable diet may lead to the deficiency of folic acid level in human body. Owing to fresh vegetables and fruits being rich in folic acid, it may be essential to reduce the incidence of APO through enough fresh vegetables and fruits intake, in the west and south of China.

The result suggested no significant difference in the frequency of *MTHFR* 1298A>C polymorphism between two groups except for the frequency of *MTHFR*1298C/C genotype in central and southern China (Table 2). It is considered that the result may be related to the larger number of specimens in the central and southern China. Therefore, folic acid intake should be increased to prevent APO in central and southern China.

In summary, no significant association was found in the frequency of *MTHFR* 1298A>C polymorphisms between patients with APO and the healthy controls in Yunnan in this study. Furthermore, we provided the important data that there was a significant correlation in the frequency of *MTHFR* 1298A>C polymorphisms between two groups in China. Therefore, it is considered that polymorphisms of *MTHFR* 1298A>C will be related to APO in China. Properly supplementation of folic acid should be recommended to prevent the occurrence of APO for the populations carrying *MTHFR *1298C/C gene.

## CONFLICT OF INTEREST

None declared.
