# Supplementary material for: Polymorphism of MTHFR 1298A>C in relation to adverse pregnancy outcomes in Chinese populations
Source: Mol Genet Genomic Med. 2019 Mar 22;7(5):e642. doi: 10.1002/mgg3.642 (PMC6503069; doi:10.1002/mgg3.642)
Supplement: Supplementary file 1 [file MGG3-7-e642-s001.docx]

**Appendix：**

Gao,J.,Wang,T.,Xiao,H.,Lou,G.Y.,Guo,L.J.,Wu,D., et al. (2015). MTHFR and MTRR gene polymorphisms involved in folate metabolism and their relations to unexplained recurrent spontaneous abortion in Henan province. *Clinical Medicine*. 35(11):1-4.

Han,Y., Pan,Y.C.,Du,Y.F.,Wan,L.ZH.,&Wang,L.(2011). Different roles of MTHFR C677T and A1298C Polymorphism in nonsyndromic cleft lip without cleft palate. *Oral Biomedicine*. 2(1):8-12.

Hu,Y.,Hou,W.,Chen,E.J.Liu,X.H.,Hou,CH.L.,&Zhang,X.H.(2011).Association of methylenetetrahy drofolate reductase A1298C polymorphisms with non-syndromic cleft lip with or without cleft palate. *Chin J Stomatol*. 46(7):394-397.

Huang,X.L.,He,Y.J.,Lu,Y.Q.,Li,Y.,&Lin,Y.X.(2015)Study on the correlation between Genetic Polymorphism of Methylenetetrahydrofolate Reductase and Threatened Abortion. *Journal of Gannan Medical University*, 35(1):41-47.

Huang,J.J.,Yue,H.Y.,&Zhang,J.(2017). Effect of polymorphisms of the methylenetetrahy- drofloate Reductase Gene(C677T, A1298C and G1793A) on Stillbirth. *J Mod Lab Med*, 32(1):72-76.

Hua,Zh.F., Huang,C.M., Lu,Y.Q.,Li,Y.,Gong,B.,&Yang,Q.(2017). Study on the correlation between IVITHFR gene polymorphism and unexplained recurrent spontaneous abortion. *Int J LabMed*, 38(1):16-17.

Jiang,Zh.E.,Cheng,Y.L.,Li,J.L.& Guo,C.H.(2003). The genetic polymorphism distribution of A1298C nudeoflde of MTHFR in patients with NDTs and its nuclens families. *Chin J Pediatr Surg*. 24(3):307-310.

Ren,J.F.,Han,X.M.,Liu,X.E.,Duan,ZH.X.,Wang.X.F.Ding,Q.L. et al. (2007). Methylenetetrahydrofolate reductase gene Polymorphism in women with recurrent pregnancy loss.Chin J Perinat Med. 10(2):80-84.

Li,J.H.(2014). Correlation between MTHFR gene polymorphism and hypertensive disorders in pregnancy. *China Medical Herald*. 11(29):11-14.

Li,X.Y.,Chen,L.G.,Guo,H.P.,&Qiang,P.(2015). Folate metabolic enzyme gene polymorphism and susceptibility to recurrent spontaneous abortion. *Contemporary Medicine*. 21(33):1-3.

Li,Q.X.,Wu,P.zh.,He,L.L.Lv,D.X.,&Fu,J.J.(2015). Relationship between folicacid metabolismrelated enzyme gene polymorphism and susceptibility of abnormal pregnancy. *Chongqing Medical Journal*. 44(10):1330-1333.

Li,X.L.,Lin,Q.SH.,Lu,Y.L. Li,Y.Tang,P.,Chen,CH.B.,et al. (2017). Investigation of the correlation of genetic polymorphism of MTHFR and MTRR and the thredtened abortion. *Chongqing Medical Journal*. 46（6）: 770-772.

Liao,Y.P.,Zhang,D.,Zhou,W.Liu,CH.Q.&Dong,H.F.(2015). Relationship between methylenetetrahy drofolate reductase and cystothione beta synthetase gene polymorphisms and Down syndrome. *Chinese Journal of Gerontology*. 35:3370-3372.

Liu,M.,Nan,X.R.,&Yuan,G.G. (2013). Relationship between genetic polymorphism of MTHFE A1298C and nonsyndromic cleft lip with or without cleft palate in Shanxi province. *Chinese Journal of Practical Stomatology*. 6(4):221-225.

Liu,N.,Yan,J.,&Yang,L. (2015). Relationship between gene polymorphism and folate metabolism among women with abnormal pregnancy history. *Chin J Farn Plann*. 23(5):318-320.

Shen,X.N.,Huang,Y.P.,Tang,Sh.H. Zhang,CH.L.,&Chen,W.S.(2009). The Relationship between the Polymorphism of MTHFR Gene and Preeclampsia. *Joural of Practical Obstetrics and Gynecology*. 25(4):236-238.

Wan,W.D.,Wang, L.J.,Zhou,X.P. Zhou,D.L.,Zhang,Q.G.,Huang,J.L.et al. (2006) Relationship between nonsyndromic deft lip with or without cleft paIate(NSCL/P)and genetic polymorphisms of MTHFR C677T and A1298C. *Chin J Plast Surg*. 22(1):8-11.

Wang,T.,Gao,J.S., Bian,X.M.,Sun,N.G.,Liu,J.T.,& Liu,X.Y.(2010). Study on the relationship of erythrocyte folate status，polymorphic variation in folate-related genes and adverse pregnancy outcomes. *J Reprod bled*. 19(1):48-52.

Xiao,L.,Liu, D.Y.,Wang,K.&Gao,J. (2012) .Study on correlation between the MTHFR Al298C

Morphisms and fetal malformations. *China Medical Herald.* 9(16):87-89.

Xiao,G.F.,Meng,X.J.,Hu,L.L.Deng,H.Y.,Zhao,Y.L.,&Wu,H.Q.(2016). Correlation analysis about folate metabolism-related genes of pregnant women vith fetal congenital defects. *Journal of Chinese Physician*. 18(7):1021-1024.

Xie,X.Y.,Zhang,Y.,Xin,L.Leng,J.H.,Xue,D. et al. (2016). The relationship of the folate metabolism related gene polymorphisms of MTHFR and MTRR with unexplained recurrent spontaneous abortion. *Tianjin Med J.* 44(10):1243-1246.

Yang,Y.,Jin,L.,Yuan,J.J. Wu,D.,Shan,Q.,Wu,L.et al. (2010). Association of Genetic Polymorphisms in Methylenetetr- ahydrofolate Reductase Gene，Plasminogen Activator In-hibitor-1 Gene with Preternl Birth and Spastic Cerebral Palsy. *J Appl Chin Pediatr*. 25(20):1580-1582.

Ye,G.CH.,Wang,D.G.,Cheng, L.Z.,Yuan,CH.L.,& Peng,J.M.(2016). Study on correlation between serum homocystine and methylenetetrahydrofolate reductase with threatened abortion. *Lab Med Clin*. 13(4):443-445.

Zheng,M.L.,Wang,G.H.,&Zhang,G.L. (2007). Relationship of plasma homocysteine(HCY)and the gene polymorphism of metabolic enzymesof the Neural Tube Defects. *Chinese Journal of Healthy Birth＆Child Care.* 13(4):158-161.

Zhu,Q.,Li,L.,Wang,T.,Jiang,W.,Ding,J.,Liu,M.J.,et al. (2016). Genetic Study of 12 SNPs involved in 11 Folate Metabolism Genes and Neural Tube Defects in Suzhou Children. *Journal of Molecular and Genetic Medicine*. 10(2):1-8.
